# Effect of the Conversion Degree on the Apparent Kinetics of Iron-Based Oxygen Carriers

**DOI:** 10.1021/acs.energyfuels.4c00928

**Published:** 2024-06-20

**Authors:** Victor Purnomo, Daofeng Mei, Ivana Staničić, Tobias Mattisson, Henrik Leion

**Affiliations:** † Division of Energy and Materials, Department of Chemistry and Chemical Engineering, Chalmers University of Technology, Göteborg 412 58, Sweden; ‡ Division of Energy Technology, Department of Space, Earth, and Environment, Chalmers University of Technology, Göteborg 412 58, Sweden

## Abstract

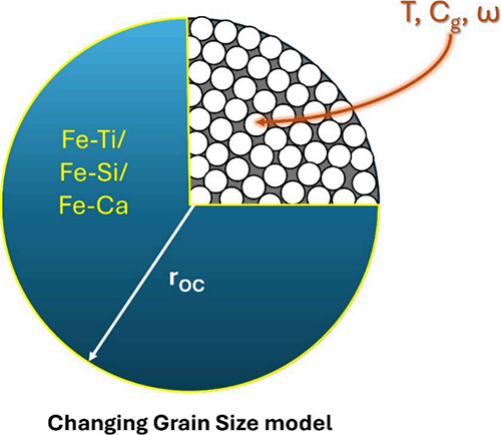

The role of the oxygen carrier is important in energy conversion processes with fluidized beds, particularly chemical looping technology. It is necessary to establish the relevant kinetics of oxygen carriers that can be applicable for various chemical looping processes. In this study, we analyzed the apparent kinetics of three iron-based oxygen carriers, namely, ilmenite, iron sand, and LD slag, during the conversion of CO, H_2_, and CH_4_ in a fluidized bed batch reactor. The effect of both the oxidation degree, presented as the mass conversion degree, and temperature was considered. The results show that the changing grain size (CGS) model is generally applicable in predicting the apparent kinetics of reactions between the investigated iron oxygen carriers and gaseous fuels even at lower oxidation degrees (3–5 wt % reduction). The activation energies of the investigated materials in the conversions of CO, H_2_, and CH_4_ obtained from the fittings of the CGS model are about 51–92, 55–251, and 72–211 kJ/mol, respectively. Both the mass conversion degree and temperature influence the reactivity of oxygen carriers in a directly proportional way, especially at temperatures higher than 925 °C. The results of this study are useful for reaction engineering purposes, such as designing a reactor, in chemical looping units, or in any other processes that use oxygen carriers as a bed material.

## 1 Introduction

Chemical looping is a promising dual fluidized bed energy conversion technique with the possibility of obtaining nearly nitrogen-free gaseous products without the need for expensive pure oxygen. This is because the oxygen-carrying bed material, usually metal oxides, captures oxygen in the air reactor and releases it in the fuel reactor, eliminating the presence of nitrogen during fuel conversion. Apart from this, the oxygen carrier plays a role in distributing heat throughout the whole reactor unit.^1^ Compared to silica sand, which is the conventional bed material in a fluidized bed combustion system,^2^ the use of an oxygen carrier can therefore increase the efficiency of the fuel conversion.^3^ This indirectly leads to a higher feasibility for the carbon capturing process since a high CO_2_ concentration in the flue gas can be expected. For this reason, the oxygen carrier has not only been utilized in pilot-scale chemical looping units but also in several oxygen carrier-aided combustion (OCAC) boilers, which have been operated both semicommercially and commercially in Sweden since 2012.^2^ This demonstrates the important role that oxygen carriers play in fluidized bed conversion, and further investigations are necessary to widen their potential application.

The production of oxygen carriers was initially done through a synthesis process in laboratories.^4^ This step together with the first utilizations of oxygen carriers in lab-scale fluidized bed units contributed to the important information on which type of metal oxide may or may not work in the targeted processes, i.e., OCAC or the already well-known chemical looping combustion (CLC). As the development progressed, it was found that iron oxides worked reasonably well as oxygen carriers.^5,6^ Not only are iron oxides reactive enough toward both gaseous and solid fuels, but they also incur a much lower production cost due to their abundant availability and negligible environmental penalty. Among the examined iron oxides, iron–titanium ore ilmenite is, as it stands, considered as a benchmark oxygen carrier^7^ since it fulfills the desired criteria for an oxygen carrier. To be specific, an oxygen carrier material is expected to be reasonably reactive to convert fuel, able to undergo multiple redox cycles, mechanically durable, abundantly available, easy to obtain, and environmentally sound.^8^

The development of oxygen carriers is still progressing in various directions as more diverse relevant research questions are formulated. For example, the use of an oxygen carrier in the currently emerging chemical looping gasification (CLG) requires different conditions from what is already known in CLC. While the main outcomes of CLC are heat and power, that of CLG is the generation of syngas, which comprises carbon monoxide and hydrogen.^9^ To obtain syngas, the amount of oxygen transferred from the air to the fuel reactor must be controlled in order to maximize the production of CO and H_2_. Obviously, the generation of CO_2_ is still expected in any case and needed in order to obtain an autothermal process, but the production will be restricted to the fuel reactor so that the eventual CO_2_ capture process will be much more efficient.^10^ The composition of the syngas itself is determined by multiple factors, such as types of fuels and oxygen carriers together with the operating conditions in the fuel reactor.^9^ This implies a challenge different from what is expected in CLC, when a high oxygen transfer capacity of an oxygen carrier is almost always desired. More details about differences between CLC and CLG can be found elsewhere.^11^

There are several strategies to limit oxygen transfer from the air to the fuel reactor in CLG. The most known one is by reducing the solid circulation rate or oxidizing gas flow.^12^ In addition, the use of certain waste-based oxygen carriers can also be plausible if they have a sufficient oxygen transfer capacity, which is most likely due to some iron content.^13,14^ These materials automatically limit the oxygen transfer from the air to the fuel reactor without having to modify the solid circulation, which can be useful in processes like CLG. Nonetheless, one should note that a low oxygen transfer capacity of an oxygen carrier often translates to a rather quick oxygen exhaustion, which leads to a highly reduced oxygen carrier. This implies several issues, such as increased risk for agglomeration, which may lead to defluidization, and decreased reactivity, also known as deactivation.^15^ Such an issue may not only be prevalent in CLG but also in other processes such as chemical looping reforming (CLR)^16^ and, even more so, chemical looping water splitting (CLWS).^16^ While both issues are well-known in the field, the latter has not been well-formulated for iron-based materials. This means that there is a lack of knowledge in explaining the relationship between the reduction degree and the reactivity, which can be covered by a kinetic study.

Multiple previous studies investigated the kinetics of oxygen carriers. Abad et al.^17^ reported the reduction kinetics toward syngas of three synthetic oxygen carriers; of them, one was an iron-based material. Some studies reported the kinetics of supported iron-based oxygen carriers for chemical looping application, either in a fixed bed system^18,19^ or TGA.^20−22^ Mendiara et al.^23^ examined the redox kinetics of an iron ore using TGA. TGA is usually used for intrinsic kinetic studies, where the focus is purely on chemical reactions.^24^ Compared to intrinsic kinetics, apparent kinetics is a more realistic approach that considers relevant external factors,^24^ which may include the effects of mass transfer and thermodynamic contributions.^25^ Hence, the main focus of apparent kinetics is not to figure out the intrinsic mechanism for a single reaction but to establish an applicable model that takes multiple relevant parameters into account. From the practical point of view, apparent kinetics can be more useful for reactor design and process modeling,^25−27^ which is the main aim of this study. There have been investigations of oxygen carriers in a fluidized bed system to derive the apparent kinetics of iron oxides.^27,28^ In this specific regard, the advantage of investigating apparent kinetics in a fluidized bed is the possibility to incorporate the effect of fluidization phenomena and the solid–gas contact pattern into the kinetic analysis. On the other hand, it is also possible to get a realistic approach by complementing intrinsic kinetics performed in TGA with relevant reactor models. However, this certainly involves more complex steps than an apparent kinetic study. In other words, the apparent kinetics was the chosen approach for this work due to its representability, as it is expected to reflect reality better, and simplicity. However, with respect to the oxidation degree, none of the apparent kinetic studies have examined Fe-based materials by considering reactions that involve iron phases with a low oxidation state, such as wüstite. This is despite such reactions being relevant for conditions in processes like CLG, CLR, and CLWS. Thus, there is a clear motivation for this apparent kinetic study to commence.

In this work, we investigated the apparent kinetics of solid–gas reactions between oxygen carriers and gaseous fuels in a fluidized bed batch reactor. The novelty lies in the fact that we considered the effect of the oxidation degree of oxygen carriers on the apparent kinetics on top of that of temperature as well as gas concentration around the particles. While most published studies focused on the reduction of Fe_2_O_3_ to Fe_3_O_4_, we aimed to obtain applicable results that also cover reduction at lower oxidation states, e.g., reduction of Fe_3_O_4_ to FeO. Since this is an apparent kinetic study, our focus is not on finding the intrinsic mechanisms that govern the reaction rate. Instead, the aim is to establish valid correlations between the reaction rate and relevant parameters (temperature and oxidation degree) that can be useful for reactor design or for similar purposes. The investigated oxygen carriers were ilmenite ore, iron sand, and LD slag, and the gaseous fuels were methane, carbon monoxide, and hydrogen. It is worth noting that all the studied materials have been examined several times as oxygen carriers in fluidized bed systems and have shown quite an acceptable performance.^13,14,29^ This certainly demonstrates the high relevance of this work to the field.

## 2 Experimental Section

### 2.1 Gaseous Fuels

Three gaseous fuels were used in this study: carbon monoxide, hydrogen, and methane. These gases were deemed to be important in a gasification process. Apart from these, syngas containing 50 vol % CO and 50 vol % hydrogen was also used in the prereduction step, which is explained in Subsection 2.4. All of the gaseous fuels were supplied by Linde Gas AB with a purity of more than 99.5%.

### 2.2 Oxygen Carriers

The ilmenite ore consisting of mostly iron and titanium was mined by Titania A/S in Norway.^30^ Iron sand is a byproduct from the copper fuming process run by Boliden AB containing mostly iron and silicon.^13^ LD slag is a byproduct from the steel converting process in SSAB (Swedish Steel), which largely comprises iron and calcium.^14^ The elemental composition of these oxygen carriers shown in Table 1 is adapted from relevant references.^13,14,30^ Note that oxygen and minor elements are excluded, so the contents do not add up to 100 wt %.

**Table 1:** Elemental Composition of Oxygen Carriers Investigated in This Study

	composition (wt %)		
element	ilmenite	iron sand	LD slag
Fe	34	35	17
Mn	0.48	0.35	2.6
Si	0.15	16	5.6
Ti	28	0.13	0.78
Ca	0.06	2.3	32
Al	0.19	2.4	0.76

Each oxygen carrier was calcined at 950 °C for 12 h in air to ensure full oxidation and then manually sieved to the size range of 125–180 μm. Based on previous publications^13,29,31^ using the same materials, the BET surface areas of freshly calcined ilmenite, iron sand, and LD slag are 0.10, 0.05, and 1.00 m^2^/g, respectively. Prior to the experimental kinetic examination, every material was first exposed to at least 3–5 redox cycles at 850 °C in the fluidized bed batch reactor to ensure stable reactivity. The reducing gas used for this procedure was 450 mL/min syngas (50% CO in H_2_) in room conditions (25 °C, 1 atm). For methane conversion, 15 g of the oxygen carrier was used, while for the other gaseous fuels, 5 g of the oxygen carrier was mixed with 10 g of quartz sand, which was assumed as an inert bed material. The latter was performed so that a fraction of the CO and H_2_ remained unconverted, which makes the observation of the reactivity change more feasible.

### 2.3 Fluidized Bed Batch Reactor Setup

**Figure 1:**
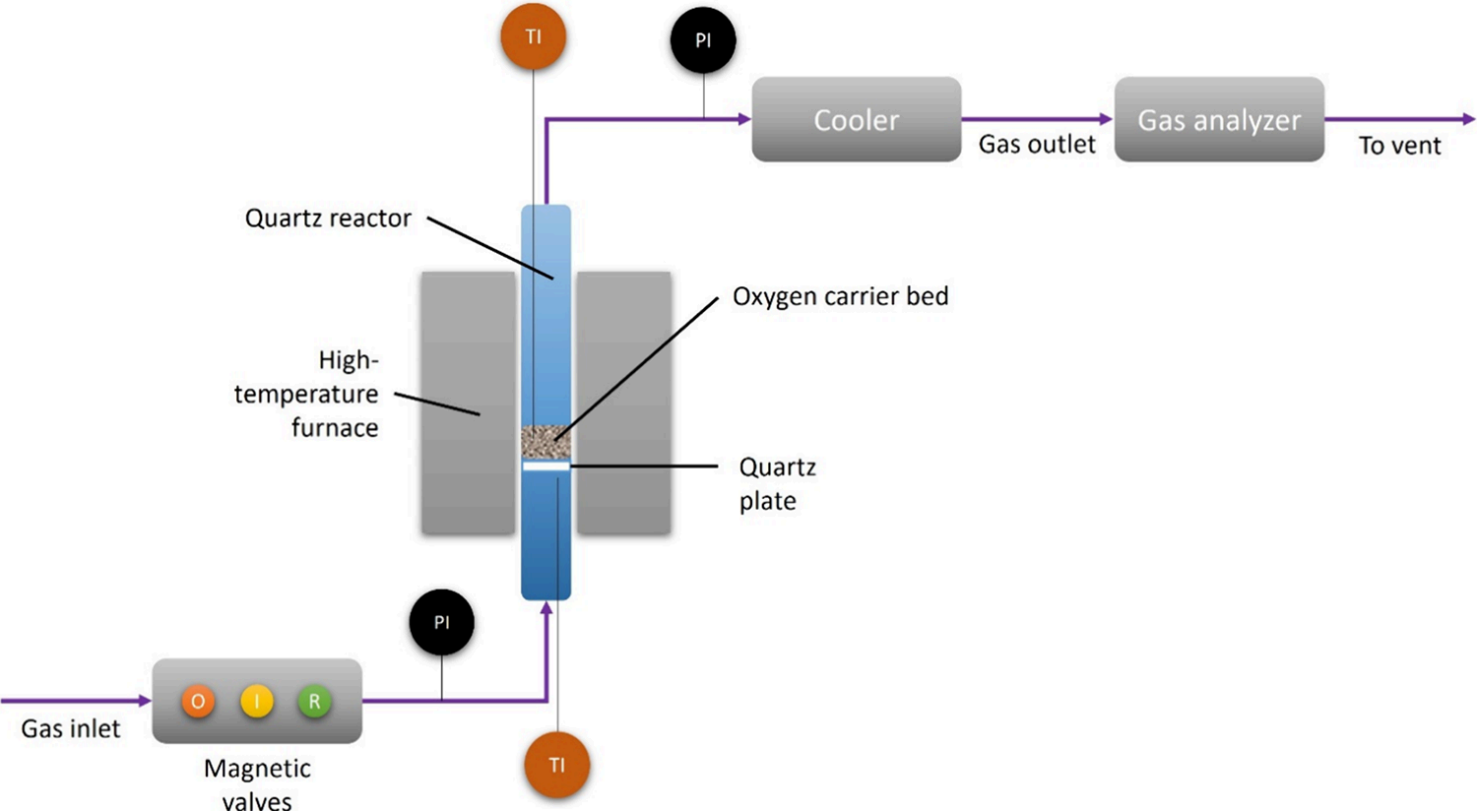
Schematic diagram of the fluidized bed batch reactor setup used in this study. PI and TI indicate the pressure and temperature indicators, respectively. The magnetic valves regulate the feeding of oxidizing (O), inert (I), and reduced (R) gases.

In the fluidized bed batch reactor setup, it was possible to mimic the oxidizing and reducing atmospheres in the air and fuel reactors, respectively, in a chemical looping setup. An inert phase was needed between the oxidizing and reducing phases to purge the remaining gas from the previous phase. Three separate magnetic valves regulated the gas feeding, see Figure 1. This setup has been previously reported.^32^

Below is a comprehensible explanation of the setup starting from left to right.

#### 2.3.1 Magnetic Valves

These were used to regulate the gas feeding into the reactor, whether it was an oxidizing (O), inert (I), or reducing (R) gas. The flow rate and composition of the gases were regulated using Brooks mass flow controllers, with a general measurement range between 700 and 1300 mL/min.

#### 2.3.2 Pressure Measurement

Pressure fluctuation is deemed effective to indicate the status of fluidization during the entire experiment. The pressure difference between the inlet and outlet lines was measured by a 20 Hz Honeywell pressure transducer. Despite having a relatively low frequency, the pressure transducer was found to work well and was able to interpret the fluidization state of the particle bed.^15^

#### 2.3.3 Reactor Dimension

A cylindrical quartz glass reactor was used with a height and inner diameter of 820 and 22 mm, respectively. Inside the reactor, about 370 mm upward from the reactor’s bottom edge lies a porous circle-shaped quartz plate. Depending on the density, usually, about 15–20 g of the particle bed was placed upon the plate, cf. Section 2.2.

#### 2.3.4 High-Temperature Furnace and Temperature Measurement

The high-temperature furnace was manufactured by ElectroHeat Sweden AB and can heat up the reactor up to 1400 °C. Nevertheless, considering the melting temperature of the quartz reactor, all the reaction temperatures in this study were below 1000 °C. The inlet and outlet connection of the reactor was tightly sealed to avoid gas leakage. During the experiments, the upper part of the reactor was wrapped with heating tape to minimize the risk of flue gas condensation. Two type-K thermocouples were used to measure the temperature of the particle bed and below the bed as a control.

#### 2.3.5 Cooler

The outlet gases were subsequently cooled down by an M&C ECP1000 condenser, which can work with a gas flow of 150 L/h or below with a total cooling capacity of 50 kJ/h at room temperature (25 °C), so that no steam entered the analyzer.

#### 2.3.6 Online Gas Analyzer

A Rosemount NGA 2000 gas analyzer measured the real-time volumetric flow rates and concentrations of the water-free flue gases after the cooling step. The repeatability of the nondispersive infrared (NDIR) analyzer module was about 1%. The sensitivity of each channel in the gas analyzer spanned from 0 to 100%, except for oxygen, whose sensitivity was within the range of 0–25%. All channels were calibrated prior to the experiment. The minimum detection capacity of CO, CO_2_, H_2_, and CH_4_ channels was around 100 ppm, while that of the O_2_ channel was around 0.1%. The H_2_ channel was particularly sensitive to interference from other gases. The gas analyzer can measure a gas flow between 500 and 1400 mL/min at a temperature between 0 and 55 °C at a pressure of 69 kPa gauge or below.

### 2.4 Methodologies

During a cycle, the oxygen carrier bed was first fully oxidized before it was prereduced with syngas (450 mL/min) to obtain the material at different oxidation degrees, which is presented as the mass conversion degree (ω). Using syngas, which is quite reactive toward iron oxygen carriers, in the prereduction step makes it possible to reach lower mass conversion degrees quickly. This last reduction step was skipped for the fully oxidized oxygen carrier (ω = 1). During the fuel conversion, gaseous fuel (450 mL/min) was introduced to the reactor in 10 pulses of 4 s each with a 60 s inert period in between. The pulsing method was chosen to enable a more accurate observation on the gradual reactivity change of the oxygen carrier as well as to make it possible to take the gas back mixing effect in the setup into account. Table 2 shows the procedure of a single cycle in detail. Note that the inert phase was introduced between each mentioned step to purge the remaining gas, which was introduced previously. Depending on the type of gaseous fuel and oxygen carrier, the ratio of fluidizing velocity to minimum fluidizing velocity (*U*/*U*_mf_) in the reactor ranges from 1.1–2.3. This applies to prereduction using syngas and fuel conversions using CO, H_2_, or CH_4_.

**Table 2:** Experimental Procedure in a Single Cycle

step	gas	volumetric flow (mL/min)	duration (s)	note
oxidation	5% O_2_ in N_2_	1000	until the OC becomes fully oxidized	until the outlet O_2_ concentration returns to 5%
inert	100% N_2_	1000	180	
prereduction	50% CO in H_2_ (syngas)	450	20–60	
inert	100% N_2_	1000	180	
fuel conversion	pure CO/H_2_/CH_4_	450	10 × 4	fed in 10 pulses
inert	100% N_2_	1000	180	

### 2.5 Data Evaluation

In this study, the effect of the mass conversion degree, i.e., the degree of solid conversion, of the oxygen carrier will be considered in the kinetic analysis. The formulas to determine the mass conversion degree of different gaseous fuels used in this study are summarized in Table 3. The integration step includes the outlet gas concentration measured during the inert phase that follows the fuel conversion step to compensate for the gas back mixing effect, see Subsection 2.4.

**Table 3:** Conversion Formulas of Different Gaseous Fuels Used in This Study^27^

fuel	eq no.	conversion formula	symbol list
syngas, 50% CO in H_2_	(1)	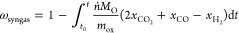	*M*_O_ = molecular weight of oxygen, *m*_ox_ = mass of the oxygen carrier at its fully oxidized state, *ṅ*_(in/out)_ = corrected^b^ molar flow (inlet/outlet), *t* = reaction time, *x*_*i*_ = molar fraction of species *i*, ω_*i*_ = mass conversion degree of the oxygen carrier upon conversion of species *i*
carbon monoxide, CO	(2)	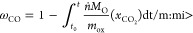	
hydrogen, H_2_^a^	(3)	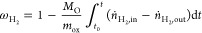	
methane, CH_4_	(4)	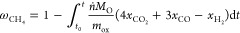	

a Since it was not possible to measure steam (the product of hydrogen conversion), a hydrogen balance was used to estimate the conversion of hydrogen.

b Corrected molar flow refers to the calculation of flow based on an elemental balance (either carbon or nitrogen). This was chosen to solve the limitation in the gas analyzer when it comes to a lower gas flow (the minimum measurable gas flow is about 0.2 L/min).

## 3 Kinetic Model Fitting

The kinetics of gas–solid reactions can be affected by various factors and can be evaluated in different devices.^33^ The focus of this study is to assess the effect of the oxygen carrier’s mass degree conversion, symbolized as ω, as well as temperature (*T*) and gas concentration (*C*_g_) around the particle on the reactivity in a fluidized bed setup.^27^ Therefore, establishing an apparent kinetic analysis seems to be more suited and essential for this purpose, considering that the experiment does not cover only a single homogeneous reaction. The results are expected to be useful for practical purposes, such as establishing the reactor design. Since most of the published studies used the degree of solid conversion α, which represents conversion of active oxygen, to correlate the solid conversion with the reactivity, there is a need to convert ω ϵ [1,0] to α ϵ [0,1] due to the different domains. This means that α indirectly represents ω. This can be expressed by the following:^34^
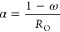
1

The oxygen carrier capacity (*R*_O_) of each oxygen carrier is theoretically defined as the ratio of the maximum amount of oxygen transferred during the reduction with fuel compared to the mass of a fully oxidized oxygen carrier.^35^ This parameter is usually determined using TGA.^36^ In practice, however, there is no absolute number for *R*_O_ as this parameter may vary depending on the experimental conditions and purposes.^37^ In the context of iron oxides, this parameter is most commonly defined based on the reduction of hematite to magnetite, so the oxygen transfer capacity of ilmenite was merely reported as 3.3 wt %.^38^ However, the materials in this study were reduced further; viz., the reduction of magnetite to wüstite, among the others, is also likely covered. Since the theoretical oxygen transfer capacity might not be suitable in this study, the oxygen transfer capacity in this study was defined based on the lowest mass conversion degrees ever reached during the experimental work regardless of the type of gaseous fuel. The assumption is that the oxygen level in the material is expected to be already exhausted or very low at this stage. For instance, the lowest mass conversion degrees reached by ilmenite during the conversions of CO, H_2_, and CH_4_ were about 0.951, 0.954, and 0.950, respectively. Therefore, as the value of α is assumed to be close to 1, the oxygen carrier capacity of ilmenite was intuitively determined as 0.050 or 5.0 wt %, see eq 1. In the same way, those of iron sand and LD slag are estimated to be 3.0 and 4.2 wt %, respectively. Note that these estimated values are likely higher than the reported values since we also consider higher reduction degrees in this study.^14,15,38^ Nonetheless, Pröll and Hofbauer^39^ suggested that the oxygen transfer capacity of natural ores and supported metal oxide materials usually lies between 2 and 10 wt %, so the estimated values seem reasonable.

The reactivity of the oxygen carrier can subsequently be expressed as follows.
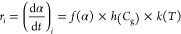
2

By using the model fitting method,^40^ here are the steps used in this study:

First, under isothermal conditions, *f*(α) can be integrated to *g*(α) by applying this formula:^41^
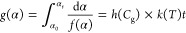
3

The experimental data can therefore be fitted to eq 3 by using different available transparent models. The most applicable models can be determined based on the linearity of the plots of *g*(α) versus time over different temperatures. The slope, d*g*(α)/d*t*, would then be the product of *h*(*C*_g_) × *k*(*T*). Wei et al.^41^ elaborated on this strategy in detail, while some relevant transparent models for solid–gas reactions have also been published.^42,43^

Table 4 shows the solid–gas kinetic models *f*(α) and *g*(α), which correspond to the derivative and integrated functions, respectively, used for the model fittings in this study. The basis assumption for these models can be found in previous publications.^42,43^

**Table 4:** Solid-State Kinetic Models Used in This Study for Fittings of the Mass Conversion Degree^42,43^

reaction model		code	*f*(α)	*g*(α)
reaction order models	first order	F1	1 – α	–ln(1 – α)
	second order	F2	(1 – α)^2^	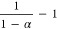
	third order	F3	(1 – α)^3^	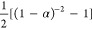
nucleation models	Avrami–Erofeyev 2	A2	2(1 – α)[−ln(1 – α)]^1/2^	[−ln(1 – α)]^1/2^
	Avrami–Erofeyev 3	A3	3(1 – α)[−ln(1 – α)]^2/3^	[−ln(1 – α)]^1/3^
	Avrami–Erofeyev 4	A4	4(1 – α)[−ln(1 – α)]^3/4^	[−ln(1 – α)]^1/4^
geometrical contraction models	contracting area	R2	2(1 – α)^1/2^	1 – (1 – α)^1/2^
	contracting volume	R3	3(1 – α)^2/3^	1 – (1 – α)^1/3^
diffusion models	1D diffusion	D1	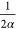	α^2^
	2D diffusion	D2	–ln(1 – α)	[(1 – α) ln(1 – α)] + α
	3D diffusion	D3	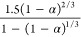	[1 – (1 – α)^1/3^]^2^
grain model	changing grain size model^a^	CGS	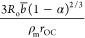	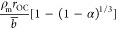

a The CGS model is specified for gas–solid reactions by assuming that a single particle comprises multiple nonporous spherical grains with the same initial grain radius.^44^ The boundary conditions are set based on the gas diffusion and concentration gradient within the particle. This model has been found to be suitable for reactions between oxygen carriers and gaseous fuels,^38^ where *b̅* is the average stoichiometric coefficient of solids, i.e., metal oxides, divided by that of the reacting gas; ρ_m_ is the molar density of gas, mol/m^3^; *r*_OC_ is the average initial radius of the oxygen carrier particles (assuming nonporous spherical particles), m.

The quality of the solid conversion model, that is, eq 3, is determined by three factors:

a) The Pearson correlation coefficient (*R*^2^), which was calculated as
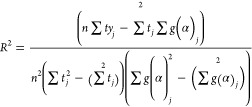
4where *n* is the data matrix size and *j* is the data index.
b) Analysis on a plot of reactivity *r*_*i*_ (eq 3) vs ω. In this step, it should be determined whether the reactivity trend is physically reasonable or not. This is because a high *R*^2^ value does not necessarily correspond to a reasonable physical model.
c) Assessment on how close the model-predicted value is to the experimental value.

The next step is to obtain the rate constant *k*(*T*) for each model from the obtained slope (eq 2). Since the slope is a product of *k*(*T*) and *h*(*C*_g_), the latter must be determined first. In this study, *h*(*C*_g_) corresponds to the molar reactant gas concentration surrounding the oxygen carrier particles. Even though the inlet gas concentration was not varied for any gaseous fuel, we assessed that the gas concentration around the particle should have an influence on the reactivity of the oxygen carriers nevertheless. Therefore, the effect of the molar gas concentration needs to be investigated as well. Assuming a first-order reaction, the boundary molar gas concentration was estimated as the logarithmic mean between inlet and outlet molar reactant gas concentrations, symbolized as *C*_g,inlet_ and outlet *C*_g,outlet_, respectively. Since the inlet gas concentration was not varied, this implies an inlet gas concentration of 100 vol %, which is equal to 44.6 mol/m^3^. The latter unit was used only in the calculation using the CGS model due to the presence of parameters like ρ_m_ and *r*_OC_.^38^ On the other hand, the outlet gas concentration was calculated as the integrated value of the measured reactant gas concentration divided by the integrated value of the total measured gas concentrations for each pulse. The gas back mixing effect was considered by including all the outlet reactant concentration in the following inert phase, see Subsection 2.5. The driving force of the molar gas concentration around the particles can therefore be formulated as
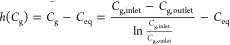
5where *C*_eq_ is the gas concentration around the particles at equilibrium. In this case, equilibrium is reached when no more observable fuel conversion takes place. At this stage, the outlet reactant concentration can be assumed to be 0 vol %, while the inlet concentration is always 100 vol %. Therefore, *C*_eq_ is taken as the average between inlet and outlet gas concentrations, that is, 50 vol % or 22.3 mol/m^3^ for the calculation using the CGS model.

In this study, however, it was later found that *h*(*C*_g_) does not change substantially, even at different temperatures. This suggests that a significant variation in gas concentrations cannot be reached without varying inlet gas concentrations in the batch reactor. Therefore, the *h*(*C*_g_) value considered in this study is the average of all the obtained *h*(*C*_g_) values within one single temperature. Hence, the *h*(*C*_g_) value is considered to be constant for each temperature.

Finally, the value of the rate constant *k*(*T*) can be determined by dividing the slope from eq 3 with that of *h*(*C*_g_) obtained from eq 5. Note that d*g*(α)/d*t* in eq 6 merely symbolizes the slope value obtained from eq 3 for each temperature and does not suggest any influence of α on *k*(*T*). Since both d*g*(α)/d*t* and *h*(*C*_g_) are constant for each temperature, this will result in a constant *k*(*T*) for each temperature as well.
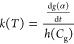
6

The obtained rate constant was then plotted against temperature according to the Arrhenius equation:
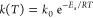
7where *k*_0_ is the pre-exponential factor, *E*_a_ is the activation energy, and *R* is the universal gas constant.

By taking the logarithmic on both sides of the Arrhenius equation (eq 7),
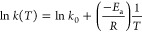
8the values of activation energy and pre-exponential factor can be obtained through linear fittings.

## 4 Results and Discussion

### 4.1 Possible Reactions during Reduction

Since both high and low oxidation degrees were considered during the investigations, it is expected that the reactions involved in this work are not only reduction of hematite to magnetite but also reduction of magnetite to wüstite. This is especially relevant to processes where oxygen carriers may experience situations, such as chemical looping gasification, reforming, and water splitting.^16^Table 5 summarizes the possible reactions taking place during the reductions and the maximum gas yield of fuel *i* (γ_*i*,max_) allowed by the thermodynamics at 900 °C. The calculations were performed using FactSage 8.2 utilizing the pure substance database.^45^

**Table 5:** Possible Reduction Reactions Taking Place during the Reductions and Their Respective Maximum Thermodynamic Gas Yield of Fuel *i* (γ_*i*,max_) at 900 °C

oxygen carrier	fuel	investigated mass conversion degree	possible reactions	γ_*i*,max_^a^ at 900 °C
ilmenite (*R*_O_ = 5.0 wt %)	CO	0.999–0.951	Fe_2_TiO_5_ + TiO_2_ + CO → 2FeTiO_3_ + CO_2_	0.999
			3Fe_2_O_3_ + CO → 2Fe_3_O_4_ + CO_2_	0.999
			Fe_3_O_4_ + CO → 3FeO + CO_2_	0.693
	H_2_	0.997–0.954	Fe_2_TiO_5_ + TiO_2_ + H_2_ → 2FeTiO_3_ + H_2_O	0.999
			3Fe_2_O_3_ + H_2_ → 2Fe_3_O_4_ + H_2_O	0.999
			Fe_3_O_4_ + H_2_ → 3FeO + H_2_O	0.739
	CH_4_	0.999–0.950	4Fe_2_TiO_5_ + 4TiO_2_ + CH_4_ → 8FeTiO_3_ + CO_2_ + 2H_2_O	0.999
			12Fe_2_O_3_ + CH_4_ → 8Fe_3_O_4_ + CO_2_ + 2H_2_O	0.999
			4Fe_3_O_4_ + CH_4_ → 12FeO + CO_2_ + 2H_2_O	0.693
iron sand (*R*_O_ = 3.0 wt %)	CO	0.999–0.977	Fe_2_O_3_ + SiO_2_ + CO → Fe_2_SiO_4_ + CO_2_	0.998
			3Fe_2_O_3_ + CO → 2Fe_3_O_4_ + CO_2_	0.999
			Fe_3_O_4_ + CO → 3FeO + CO_2_	0.693
	H_2_	0.995–0.971	Fe_2_O_3_ + SiO_2_ + H_2_ → Fe_2_SiO_4_ + H_2_O	0.998
			3Fe_2_O_3_ + H_2_ → 2Fe_3_O_4_ + H_2_O	0.999
			Fe_3_O_4_ + H_2_ → 3FeO + H_2_O	0.739
	CH_4_	0.999–0.983	4Fe_2_O_3_ + 4SiO_2_ + CH_4_ → 4Fe_2_SiO_4_ + CO_2_ + 2H_2_O	0.998
			12Fe_2_O_3_ + CH_4_ → 8Fe_3_O_4_ + CO_2_ + 2H_2_O	0.999
			4Fe_3_O_4_ + CH_4_ → 12FeO + CO_2_ + 2H_2_O	0.693
LD slag (*R*_O_ = 4.2 wt %)	CO	0.998–0.958	3Fe_2_O_3_ + CO → 2Fe_3_O_4_ + CO_2_	0.999
			Fe_3_O_4_ + CO → 3FeO + CO_2_	0.693
	H_2_	0.995–0.974	3Fe_2_O_3_ + H_2_ → 2Fe_3_O_4_ + H_2_O	0.999
			Fe_3_O_4_ + H_2_ → 3FeO + H_2_O	0.739
	CH_4_	0.999–0.977	12Fe_2_O_3_ + CH_4_ → 8Fe_3_O_4_ + CO_2_ + 2H_2_O	0.999
			4Fe_3_O_4_ + CH_4_ → 12FeO + CO_2_ + 2H_2_O	0.693

a

**Figure 2:**
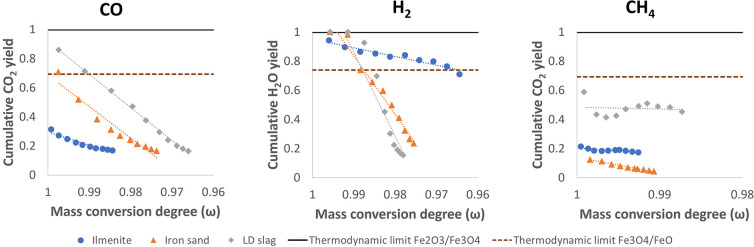
Observed maximum gas yields for ilmenite (blue), iron sand (orange), and LD slag (gray) at 900 °C for three fuels presented in panel CO, H_2_, and CH_4_. The theoretical gas conversion limitations of Fe_2_O_3_/Fe_3_O_4_ and Fe_3_O_4_/FeO are shown as solid and dotted lines, respectively.

It is necessary to establish whether the reactions presented in Table 5 are kinetically or thermodynamically limited. This is assessed by comparing the maximum yield observed during the experiment to the theoretical maximum yield allowed by thermodynamics. The thermodynamic limit at 900 °C for each individual reaction presented in Table 5 and the highest gas yield, i.e., CO_2_ gas yield for conversions of CO and CH_4_ and H_2_O gas yield for conversion of H_2_, observed during experiments performed at 900 °C is summarized in Figure 2. With respect to the conversion of Fe_2_O_3_ to Fe_3_O_4_ (as well as Fe_2_TiO_5_ + TiO_2_/FeTiO_3_ and Fe_2_O_3_ + SiO_4_/Fe_2_SiO_4_), the maximum thermodynamic limit is always above 99.8%. Thus, it is evident that these limits are not reached under experimental conditions for CO and CH_4_. For H_2_, on the other hand, an almost complete conversion was achieved at high mass conversion degrees (ω > 0.995), and thus, the corresponding experimental points are close to thermodynamic equilibrium. Due to this, the reactivity of iron sand and LD slag during H_2_ conversion was only examined at mass conversion degrees lower than 0.995.

Another thermodynamic limit that might need to be considered is Fe_3_O_4_/FeO. As shown in Figure 2, some of the experimental values, especially in the case of reactions with H_2_, are found to be above this limit. However, it should be noted that the reactions listed in Table 5 may not likely happen at the same time. The most logical and likely scenario is that conversion of Fe_2_O_3_ to Fe_3_O_4_ takes place at a higher mass conversion degree followed by reduction of Fe_3_O_4_ to FeO at lower mass conversion degrees.^46^ Therefore, this theoretical limit is not quite relevant for this work, especially at higher mass conversion degrees. Note that the oxygen carriers were always fully oxidized prior to the apparent kinetic investigation. Thus, the more applicable limit for this work would be that of Fe_2_O_3_/Fe_3_O_4_.

### 4.2 Determination of Kinetic Parameters

**Figure 3:**
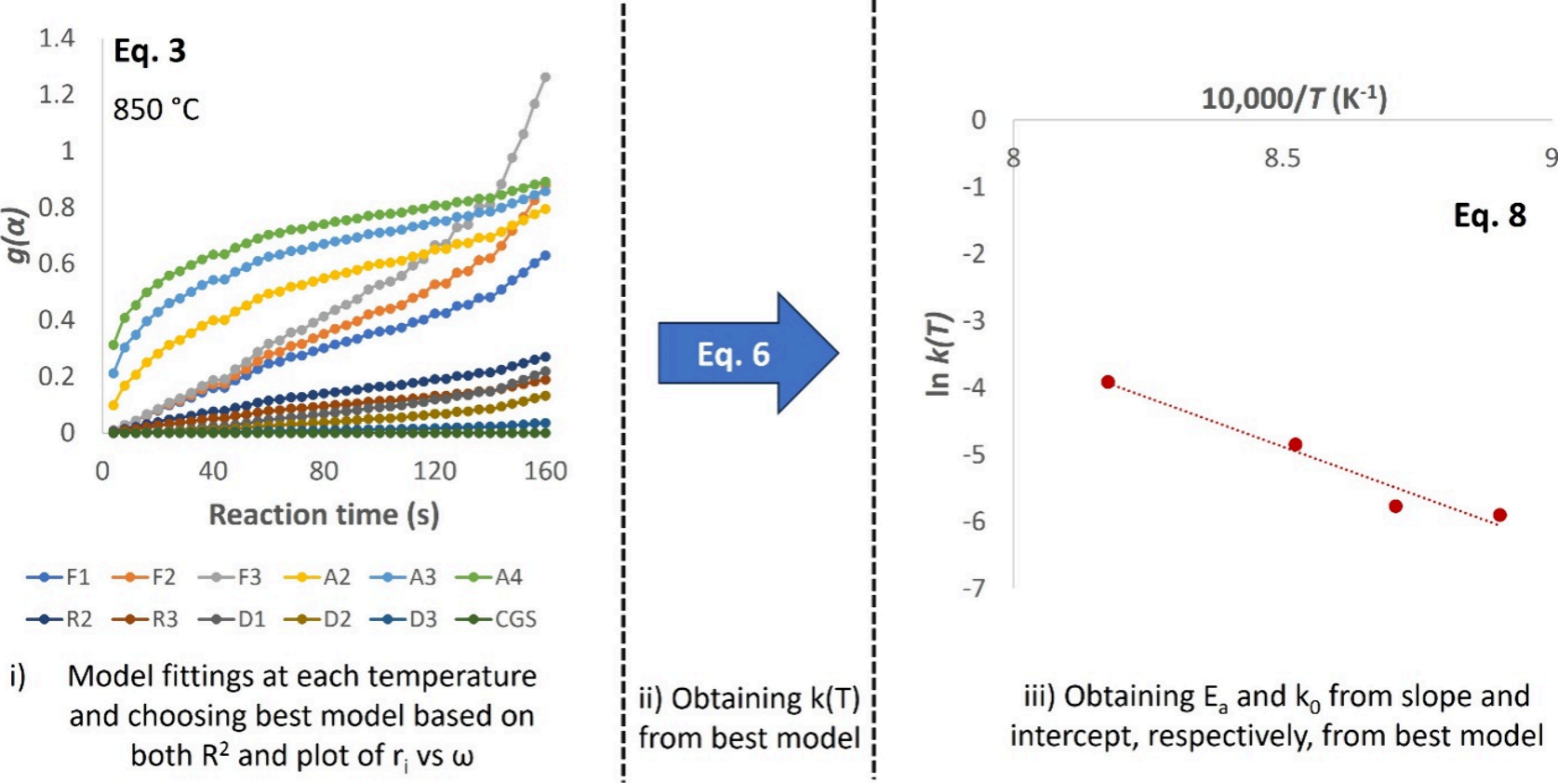
Illustration of model fittings for reactions between ilmenite and methane. In this figure, the first step is illustrated at 850 °C. Every point in (i) represents a pulse of fuel in the experiment.

The fitting steps for various transparent solid–gas reaction models are elaborated in Section 3. Figure 3 demonstrates how these steps were performed on reactions between ilmenite and methane. The best model chosen in this study is the one that fulfills these conditions:

a) showing the highest linearity in step (i) based on the Pearson correlation coefficient, see eq 4,^41^ over different temperatures,
b) showing a physically reasonable trend in the plot of reactivity, symbolized as *r*_*i*_, (eq 2) vs the mass conversion degree, symbolized as ω, and
c) predicting the closest reactivity to the experimental data.

The chosen model was the basis to calculate the rate constant *k*(*T*) in step (ii) as well as the activation energy *E*_a_ and pre-exponential factor *k*_0_ in step (iii).

Models with the highest Pearson coefficient correlation seem to vary depending on the types of oxygen carriers and gaseous fuels. Table 6 shows models with the highest correlation coefficient *R*^2^ for each oxygen carrier–gaseous fuel pair. Note that the investigated temperatures for ilmenite–CO and iron sand–H_2_ are slightly different from those of the others.

**Table 6:** Kinetic Parameters of Models with the Highest *R*^2^ for Each Oxygen Carrier–Gaseous Fuel Pair

oxygen carrier	gaseous fuel	investigated temperatures (°C)	best model	*R*^2^	*k*_0_ (s^–1^)	*E*_a_ (kJ/mol)
ilmenite (*R*_O_ = 5.0 wt %)	CO	850, 900, 950, and 975	A3	0.9394	7.34	59.4
	H_2_	850, 875, 900, and 950	R2	0.9896	8.7 × 10^8^	235
	CH_4_	850, 875, 900, and 950	R2	0.9648	1.3 × 10^8^	227
iron sand (*R*_O_ = 3.0 wt %)	CO	850, 875, 900, and 950	R3	0.9387	0.73	51.7
	H_2_	850, 875, 900, 925, and 950	D2	0.9697	1.7 × 10^5^	154
	CH_4_	850, 875, 900, and 950	D1	0.9806	178	109
LD slag (*R*_O_ = 4.2 wt %)	CO	850, 875, 900, and 950	D1	0.9077	9.78	64.8
	H_2_	850, 875, 900, and 950	D3	0.9765	19.3	95.1
	CH_4_	850, 875, 900, and 950	D1	0.9894	4,012	141

**Figure 4:**
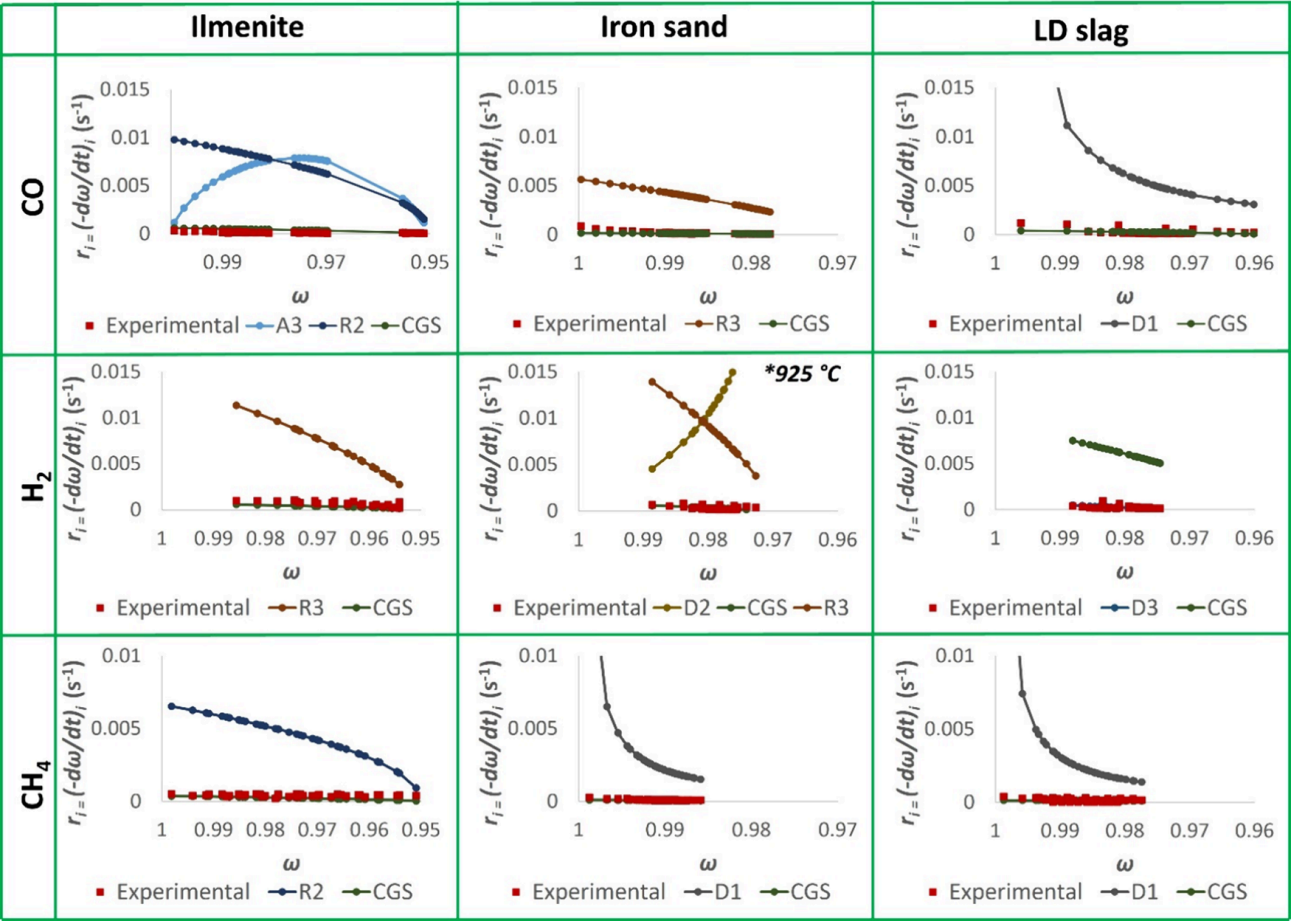
Plots of reactivity, *r*_*i*_, vs mass conversion degree, ω, based on the experimental values as well as the predicted values from models with the highest correlation coefficient and CGS at 950 °C. Note that iron sand–H_2_ was performed at 925 °C.

However, it turns out that a high *R*^2^ value is not sufficient. Other criteria must also be fulfilled in order to establish a reliable model fitting. To illustrate this, Figure 4 shows the plot of reactivity vs the mass conversion degree at 950 °C based on the experimental values as well as the predicted values from

(i) the model with the highest correlation coefficient *R*^2^ (see Table 6),
(ii) another model with the next highest *R*^2^ that shows a reasonable trend, i.e., decreasing reactivity, in the case that the model in (i) shows an unreasonable reactivity trend, and
(iii) CGS, which is commonly used for kinetic study of oxygen carriers.^27,38,47,48^

For iron sand–H_2_, the results from the experiment at 925 °C are shown instead of that at 950 °C. This is due to the bed defluidization at lower mass conversion degrees at 950 °C.

The model fittings in Figure 4 demonstrate that a model fitting with a high *R*^2^ value does not necessarily indicate that the model is applicable. There are two types of issues observed in this work.

(i) Unreasonable Reactivity Trend. Two applicable examples for this issue are ilmenite–CO and iron sand–H_2_, whose models with the highest *R*^2^ are A3 and D2, respectively. In both cases, the respective model predicts an increase followed by a sharp decrease in reactivity, which is far away from the real trend. The initial thought was to solve this by picking another model with the second highest *R*^2^ that shows a reasonable trend.
(ii) Significantly Overestimated Reactivity. Figure 4 clearly shows that even if a model has an acceptable *R*^2^ and shows a reasonable reactivity trend, it may overestimate the reactivity. Further analyses prove that the CGS model is, in turn, the most applicable model, as this model is able to predict reactivity values that are the closest to the experimental ones in most cases. The exception is LD slag–H_2_, where the D3 model with the highest *R*^2^ fits the experimental data better than CGS. This might be attributed to the complex composition of LD slag, so diffusion may be an important mechanism in this case. Still, for the sake of practicality, it can be deduced that the CGS model generally works well for establishing apparent kinetics of the oxygen carrier–gaseous fuel. The CGS model has a similar formula to the R3 model (and hence the same *R*^2^), but this model also considers parameters such as the grain size, stoichiometric coefficient, and gas molar density. None of the other models take these factors into account; therefore, they tend to overestimate the reactivity values despite the high correlation coefficient (*R*^2^). Table 7 shows the coefficient correlation *R*^2^ as well as the obtained kinetic parameter for each oxygen carrier–gaseous fuel pair when using the CGS model.

**Table 7:** Kinetic Parameters for Each Oxygen Carrier–Gaseous Fuel Pair Obtained Using the CGS Model

oxygen carrier	gaseous fuel	investigated temperatures (°C)	*R*^2^	*k*_0_ (s^–1^)	*E*_a_ (kJ/mol)
ilmenite *(R*_O_ = 5.0 wt %)	CO	850, 900, 950, and 975	0.8809	0.003	91.6
	H_2_	850, 875, 900, and 950	0.9895	1 × 10^5^	251
	CH_4_	850, 875, 900, and 950	0.9507	137	211
iron sand (*R*_O_ = 3.0 wt %)	CO	850, 875, 900, and 950	0.9387	2.2 × 10^–5^	51.5
	H_2_	850, 875, 900, 925, and 950	0.9662	6.6	161
	CH_4_	850, 875, 900, and 950	0.9395	2.3 × 10^–5^	72.4
LD slag *(R*_O_ = 4.2 wt %)	CO	850, 875, 900, and 950	0.8719	4 × 10^–4^	74.8
	H_2_	850, 875, 900, and 950	0.9479	8 × 10^–4^	55.5
	CH_4_	850, 875, 900, and 950	0.9468	0.004	122

According to Abad et al.,^38^ the activation energies of the reduction of ilmenite using the CGS model in conversions of CO, H_2_, and CH_4_ are about 80.7, 65.0, and 135.2 kJ/mol, respectively. The corresponding activation energy values of ilmenite obtained from this study are therefore generally higher than the reported values. However, the previous study did not take reduction from magnetite to wüstite into account, while it is known that the reactivity of the oxygen carrier is generally low in this region. This may explain why the reactions in this study seem to require a higher activation energy.

### 4.3 Analysis on the Apparent Kinetic Mechanisms

As mentioned in Section 1, the main purpose of this study is to establish applicable apparent kinetics for reactions between the investigated oxygen carriers and gaseous fuels. Based on different criteria, CGS is deemed the most reliable model for most reactions between oxygen carriers and gaseous fuels in this study. However, some analytical discussion of the apparent kinetic mechanisms can also be made.

**Figure 5:**
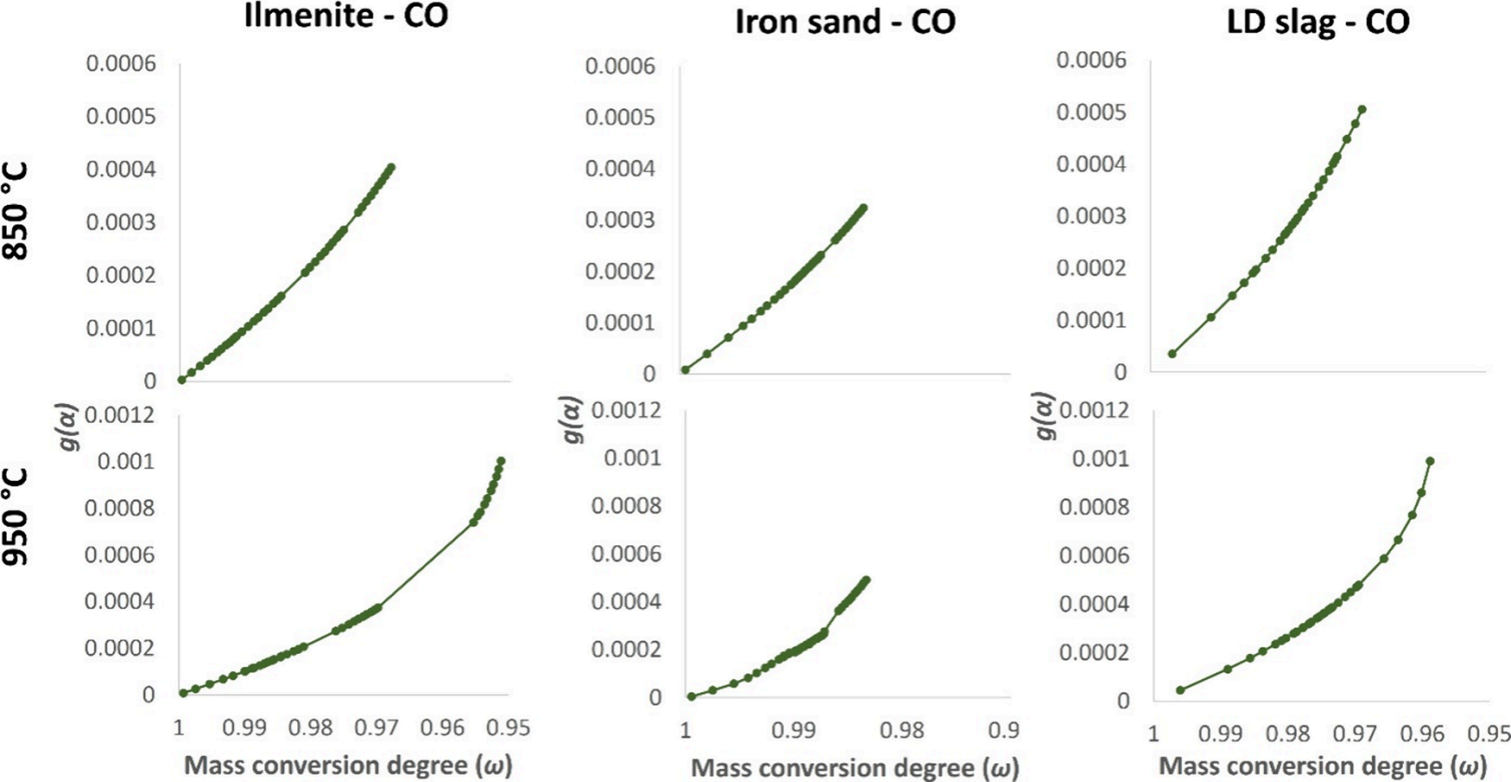
Plots of *g*(α) vs mass conversion degrees (ω) for reactions with CO at 850 and 950 °C using the CGS model.

For a start, Table 6 shows that the fittings of reactions with CO show a lower Pearson correlation coefficient (*R*^2^) compared to those with H_2_ and CH_4_. This is because the plots of *g*(α) versus time for reactions with CO do not show a continuous linear line but rather different stages of correlation at different time periods. However, such plots cannot be necessarily used to demonstrate the presence of relevant mechanisms in this study. This is because we did not perform an uninterrupted series of pulses from the fully oxidized state (ω = 1). Instead, the presence of a prereduction step (see Table 2) means a noncontinuous series of pulses. A more representative graph would be the plot of *g*(α) versus the mass conversion degree (ω) as the value of ω can be easily determined and compared even in the case of interruption between series of pulses. Note that choosing α as the *x*-axis may be equally representative in some cases, but the mass-based parameter ω is preferred in this study. Figure 5 below shows plots of *g*(α) vs ω for reactions between CO and the three materials using the CGS model at 850 and 950 °C. Every point in Figure 5 represents a single 4 s pulse of fuel in the experiment, see Subsection 2.4 for more details.

Figure 5 shows different *g*(α) trends at different temperatures, which are likely due to different reaction mechanisms. At 850 °C, only a single line is observed, so the conversion of CO using any oxygen carrier is likely governed by a single mechanism in this case. However, there seem to be two different *g*(α) trends and, therefore, two different reaction mechanisms at 950 °C. This demonstrates that the mechanism transition may occur at different mass conversion degrees over different temperatures. Similarly, Wei et al.^41^ have also reported a mechanism transition on the reactions between a hematite ore and hydrogen at a higher temperature. At higher mass conversion degrees, the chemical reaction is usually the rate-determining mechanism; this is also one of the relevant assumptions for the CGS model.^38^ At lower mass conversion degrees, another reaction may take place, and this implies a different reaction mechanism. For instance, the outward migration of iron phases to the particle surface during reduction^49^ creates a chemically distinct iron layer,^50^ which implies formation of the Fe/FeO interface.^15,51^ As a result, the diffusion rate of fuel into the particle becomes much slower, so the reaction is likely controlled by diffusion at this stage.^52^ All in all, this suggests that it is not unlikely that different mechanisms take place in the solid–gas reactions covered in this study due to different reactions. However, as the main aim of this study dictates, the focus of this work is to present an applicable apparent kinetics of each solid–gas reaction, i.e., each oxygen carrier–gaseous fuel pair, which is already discussed in Subsection 4.2.

### 4.4 Effect of the Mass Conversion Degree and Temperature on Reactivity

**Figure 6:**
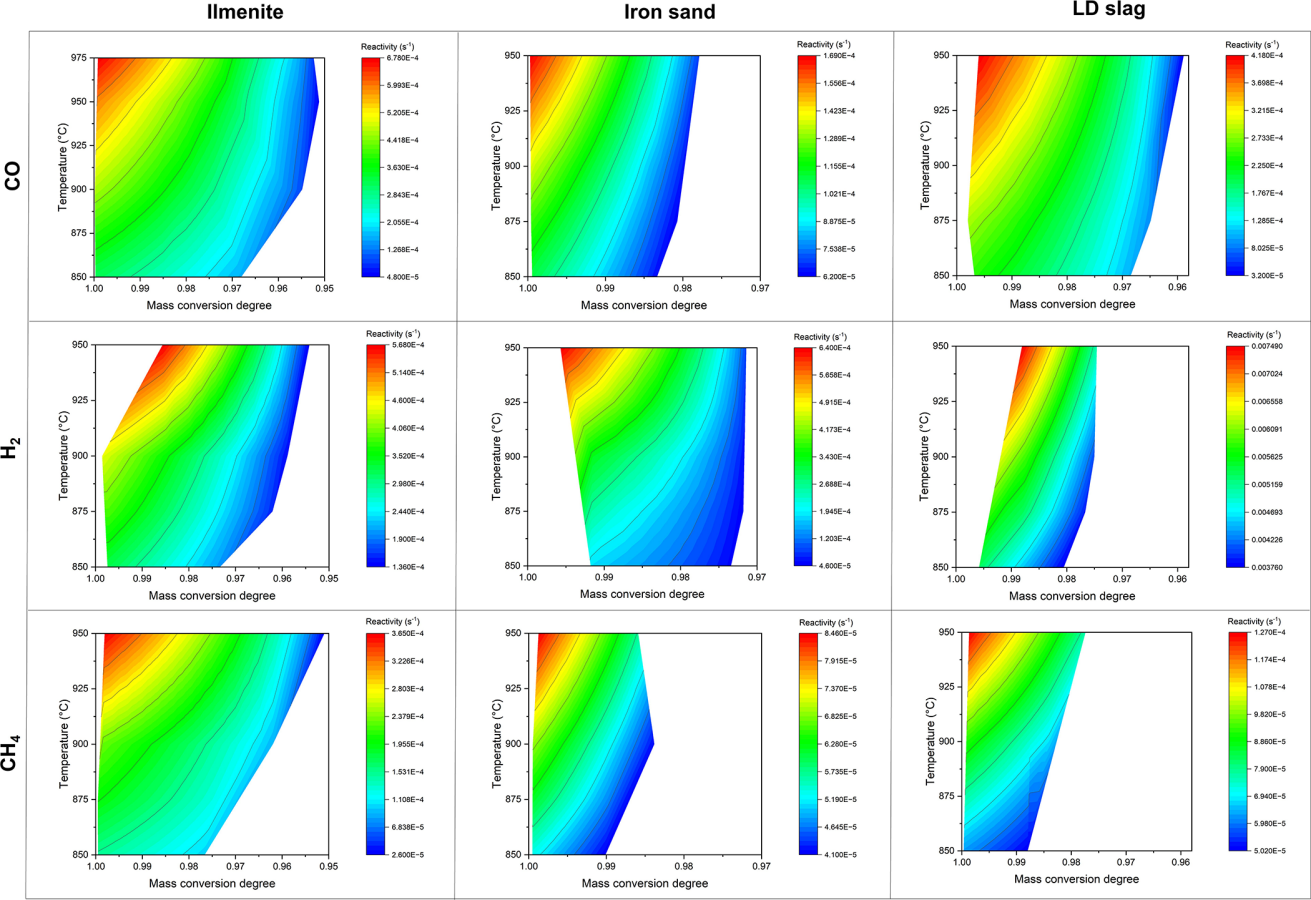
Contour plots of reactivity vs temperature and mass conversion degree.

Since the inlet gas concentration was not varied in this study, the focus is to evaluate the effect of the mass conversion degree and temperature on the reactivity of the oxygen carrier. The CGS model is chosen as the basis of the discussion for this purpose. Figure 6 shows contour plots that visualize how the mass conversion degree and temperature influence reactivity. Here, the *x*- and *y*-axes of the graph are the mass conversion degree and temperature, respectively, while the color scales indicate the spectrum of reactivity, which is obtained from eq 2. Since the molar gas concentration around the oxygen carriers’ particle (calculated using eq 5) was found to be relatively stable independent of the mass conversion degree, the effect of molar gas concentration on the reactivity is not represented in the graph. The mass conversion degree scale is adjusted according to the oxygen transfer capacity of each material, see Section 3. Note that the temperature scale of the ilmenite–CO pair is different from the others due to different investigated reaction temperatures, see Subsection 4.2.

An obvious effect of the mass conversion degree on reactivity with a positive correlation can be observed in Figure 6. This is more pronounced at temperatures higher than 925 °C, where the reactivity of all oxygen carriers clearly decreases at lower mass conversion degrees. This applies to all investigated oxygen carrier–gaseous fuel reactions. Such an influence at a low temperature, e.g., 850 °C, is not always apparent, as can be seen with some cases, such as iron sand–H_2_, iron sand–CH_4_, and LD slag–CH_4_. However, this can simply be due to color grading and does not necessarily indicate that there is no significant reactivity change at such a low temperature. On the other hand, the effect of temperature is obvious in all cases: the higher the temperature, the higher the reactivity. This is in line with the generally accepted knowledge that the reaction rate is directly proportional to the temperature.^53^

### 4.5 Implication to Chemical Looping Application

Since the apparent kinetic analysis done in this study was performed in a fluidized bed instead of a fixed bed or a TGA, the results are relevant to any chemical looping process and even any other fluidized bed processes that use an iron-based oxygen carrier as the bed material. In general, it can be assumed that the CGS model is one of the most plausible kinetic models for reactions between iron oxygen carriers and gaseous fuels, even when lower oxidation degrees are considered in the process. Our analysis indicated a few mechanisms that may determine the reaction rate at different oxidation degrees, but this is not the main aim of this study. Instead, the results successfully demonstrate that the CGS model is applicable for apparent kinetic analysis of reactions between iron oxygen carriers and various gaseous fuels even at lower mass conversion degrees (3–5 wt % reduction). Therefore, it is recommended that this model be used for various reaction engineering applications, such as reactor design.

Furthermore, it is clear that both the mass conversion degree and temperature influence the reactivity of the oxygen carrier. The effect of the mass conversion degree seems significant at temperatures higher than 925 °C. In processes like chemical looping combustion, the effect of the mass conversion degree may not be so crucial, as the oxygen carrier is usually only reduced to a moderate level. However, in processes like chemical looping gasification, reforming, or especially water splitting, less oxygen transfer from the air reactor (AR) to the fuel reactor (FR) is required to establish partial fuel oxidation. This likely leads to a lower oxidation level of the oxygen carrier, so the effect of the mass conversion degree on reactivity may thus not be ruled out. The results of this study can therefore be useful for such processes.

## 5 Conclusions

Considerable experimental work and analysis have been done to evaluate the apparent kinetics of three iron oxygen-carrying materials toward three different gaseous fuels. The effect of the mass conversion degree and temperature on the reactivity was considered in the model fittings. The results demonstrate that the changing grain size (CGS) model, which is commonly used in previous kinetic studies of oxygen carriers, is applicable to predict the reactivity of iron oxygen carriers toward all of the investigated gaseous fuels in a large conversion range. This is despite the various mechanisms that govern the reaction rate at different oxidation levels. According to this model, the activation energies of the investigated materials in the conversions of CO, H_2_, and CH_4_ even at lower mass conversion degrees (3–5 wt % reduction) are about 51–92, 55–251, and 72–211 kJ/mol, respectively. Both the mass conversion degree and temperature clearly influence the reactivity of oxygen carriers, especially at temperatures higher than 925 °C. These results are useful for reaction engineering purposes, such as designing a reactor in any chemical looping process as well as any other technology that uses an iron oxygen carrier as bed materials.

## ABBREVIATIONS

AR: air reactor
*b̅*: average stoichiometric coefficient of metal oxides divided by that of the reacting gas
*C*_g_: molar gas concentration around particles (vol % or mol m^–3^ for the CGS model)
CGS: changing grain size
CLC: chemical looping combustion
CLG: chemical looping gasification
CLR: chemical looping reforming
CLWS: chemical looping water splitting
*E*_a_: activation energy (kJ mol^–1^)
FR: fuel reactor
*f*(α): kinetic function of solid conversion in derivative forms
*g*(α): kinetic function of solid conversion in integrated forms
*h*(*C*_g_): reactivity as a function of the molar gas concentration around particles
*j*: data index in eq 6
*k*_0_: Arrhenius pre-exponential factor (m^3^ mol^–1^ s^–1^)
*k*(*T*): temperature-based rate constant (m^3^ mol^–1^ s^–1^)
LD: Linz–Donawitz
*M*_O_: molecular weight of oxygen (16 g mol^–1^)
*ṁ_o_*: stoichiometric amount of oxygen
*n*: matrix size in eq 6
*ṅ*: molar flow (mol s^–1^)
OCAC: oxygen carrier-aided combustion
*r*: reactivity of the oxygen carrier (s^–1^)
*R*_O_: maximum observable oxygen transfer capacity (wt %)
*r*_OC_: average initial radius of the oxygen carrier particles (m)
*t*: time
*T*: temperature
*X*: degree of solid conversion
*x*: molar fraction
α: solid conversion fraction
γ_*i*,max_: maximum gas yield of fuel *i* allowed by thermodynamics
ω: mass conversion degree
ρ_m_: molar density of gas (mol m^–3^)
